# The effect of fruit extracts containing phenolic compounds on the survivability of *Streptococcus mutans*: an in vitro study

**DOI:** 10.1007/s40368-025-01156-w

**Published:** 2026-01-19

**Authors:** Karin Zornitzki, Daniel Z. Bar, Avital Shmerling, Sigalit Blumer, Gisela Berenstein Ajzman

**Affiliations:** Department of Pediatric Dentistry, The Maurice and Gabriela Goldschleger School of Dental Medicine, Gray Faculty of Medical and Health Sciences, Tel Aviv University, Tel Aviv, Israel

**Keywords:** Dental caries, *Streptococcus mutans*, Fruit extracts, Cranberry, Raisins, Cherry, Pomegranate, Prevention

## Abstract

**Purpose:**

To examine the dose–response of various fruit extracts on the growth of *S. mutans* as a primary cariogenic bacterium in the oral microbiota and to determine the minimal effective concentration that inhibits its growth.

**Methods:**

Fruit extracts of dried cranberries, cherries and raisins, as well as fresh pomegranate peel extract and pomegranate juice, were prepared and serially diluted with sterile deionized water to known concentrations of 1, 5, and 20 μg/μl. The effect of each fruit extract on the growth inhibition of *S. mutans* over 6 h was determined. Sterile deionized water served as a negative control and chlorhexidine (0.2%) served as a positive control.

**Results:**

Extracts of raisins, cranberries, and cherries, as well as pomegranate peel extract and juice, inhibited *S. mutans* growth in a dose-dependent manner. Significant inhibition at 6 h was observed for raisin, cranberry and cherry extracts at 5 and 20 μg/μl, as well as for pomegranate peel extract and juice at 1 – 20 μg/μl (all *p* < 0.05 vs. negative control). Among the highest concentrations tested, pomegranate peel extract (20 μg/μl) demonstrated the strongest inhibition, statistically significantly surpassing raisin, cranberry and cherry extracts, and pomegranate juice. Notably, the inhibitory effect of pomegranate peel extract did not differ from chlorhexidine, indicating that at this concentration it is as effective as the positive control.

**Conclusions:**

Among fruit extracts, pomegranate peel extract (20 μg/μl), was the most effective inhibitor of *S. mutans *in vitro, matching the activity of chlorhexidine (0.2%), and could be further developed as a natural alternative for oral health applications.

## Introduction

Dental caries is among the most common chronic diseases affecting children and adults globally, posing significant challenges to public health and to the well-being of affected individuals and their families (WHO 2022). Untreated caries lesions of permanent teeth remained the most prevalent condition among 371 diseases and injuries assessed in the Global Burden of Disease database in 2021, with over 2.2 billion cases worldwide (Bernabe et al. 2025).

Dental caries is a multifactorial disease, with significant roles attributed to host factors, such as dietary habits, oral hygiene, salivary buffering capacity, tooth morphology, genetic predisposition, and fluoride exposure (Pitts et al. 2017). While at least 14 genera were identified in sub-gingival and supra-gingival plaque (Duran-Pinedo 2021), not all species within these genera actively contribute to cariogenesis (Benítez-Páez et al. 2014; Solbiati and Frias-Lopez 2018). Additionally, microbial composition can vary with caries lesion stage, lesion type, tooth type, and dentition phase (Dinis et al. 2022b; Kressirer et al. 2018).

In children, *Streptococcus mutans* (*S. mutans*) and *Streptococcus sobrinus* (*S. sobrinus*) are the main etiologic bacteria in the initiation of caries lesion development, while *Candida albicans* and *Lactobacillus *spp. also contribute, particularly in early childhood, through their presence in dental plaque (Berkowitz 2006; Sulyanto et al. 2024; Takahashi and Nyvad 2011). High sugars and high carbohydrate diets contribute to the growth and activity of *S.* *mutans *and *Lactobacillus* spp., exacerbating the progression of caries risk in affected children (Pidamale et al. 2024).

Some polyphenol-rich fruits, such as pomegranate, berries (strawberry, raspberry, blackberry, and cranberry), cherries and grapes/raisins, are considered protective against the development of caries lesions. Polyphenols in fruit and tea inhibit bacteria through multiple, complementary actions: they disrupt or permeabilize membranes, impairing ion homeostasis; bind and inhibit key enzymes (e.g., fatty acid synthase components) and interfere with nucleic acids; they also chelate metals and modulate redox balance, contributing to oxidative damage. Many polyphenols attenuate virulence by blocking quorum sensing and suppressing biofilm matrix formation (Farkash et al. 2019; Flemming et al. 2021; Olchowik-Grabarek et al. 2022; Sun et al. 2024). These properties could help develop functional foods, beverages, or oral hygiene products such as mouthwashes or toothpastes enriched with specific fruit extracts to reduce caries experience in children. Therefore, the present study aimed to examine, in vitro, the dose–response effects of various fruit extracts on the growth of *S. mutans* as a primary cariogenic bacterium in the oral microbiota, and to determine the minimal effective concentration required to inhibit its growth.

## Materials and methods

The effect of fruit extracts on *S. mutans* growth was evaluated in vitro model of dental caries by employing the dilution tube method, according to the Clinical and Laboratory Standards Institute (CLSI) guidelines (CLSI M100™ Performance standards for antimicrobial susceptibility testing 34th edition 2024). Prior to conducting the experiment, three pilot studies were carried out to improve the precision of the results.

### Preparation of fruit extracts

Store-bought dried cranberries, cherries, and raisins with no added sugar (all infused with apple juice before drying), and fresh pomegranate peel (10 g each) were crushed separately using a mortar and pestle and soaked in 20 ml of deionized water for 30 min. Pomegranate juice was extracted from10 g of pomegranate seeds using a mortar and pestle. The soaked fruit and the pomegranate juice were centrifuged to separate the liquid fruit extract from the solid fruit components. The liquid fruit extracts and the pomegranate juice were then lyophilized. Following lyophilization, each dried fruit extract powder was serially diluted with deionized water to achieve a concentration of 1, 5, and 20 μg/μl of fruit extract. Then, 0.5 ml of Brain Heart Infusion Broth (BHI; Cat# 2,082,495, BD, Franklin Lakes, NJ, USA) and 0.5 ml deionized water were added to each fruit extract dilution. BHI broth is a nutrient-rich medium commonly used for oral bacteria (Chapter 2 techniques for oral microbiology 2015).

### Bacterial strain and culture conditions

*S. mutans* strain ATCC UA159 was kindly provided by Prof Gilad Bachrach (The Hebrew University of Jerusalem, Israel). The strain was cultured according to American Type Culture Collection (ATCC) guidelines (*S. mutans* Clarke) and confirmed as *S. mutans* using targeted polymerase chain reaction (PCR), DNA sequencing, and protein mass spectrometry.

For the experiments, *S. mutans* spp. were grown in BHI broth and diluted to an optical density at 600 nm (OD_600_) of 0.4 using fresh BHI. Ten microliters of the bacterial suspension was added to 1 ml test tubes containing known concentrations of fruit extract in BHI. The mixtures were vortexed, and 200 μL of each was transferred to a 96-well micro-titer plate, with each concentration tested in triplicate.

Bacteria suspended in sterile deionized water served as the negative control, and bacteria exposed to 0.2% chlorhexidine served as the positive control.

Plates were incubated at 37 °C with continuous shaking for 12 h in a microplate reader (BioTek Synergy HTX, Agilent, Santa Clara, CA, USA). Bacterial growth was quantified by measuring OD_600_ every 10 min. A reduction in OD_600_ relative to the negative control (deionized water) indicated growth inhibition. Inhibition without complete reduction in OD_600_ was interpreted as bacteriostatic, while OD values comparable to the positive control (chlorhexidine 0.2%) were considered indicative of a bactericidal effect (Jenkins et al. 1988; Karpiński and Szkaradkiewicz 2015). As growth saturation was observed at 6 h, growth curves are presented up to this time point.

### Statistical analysis

The mean and standard deviation of each OD reading were calculated; OD readings of the different fruit extracts were compared by two-tailed t test. To correct for multiple hypotheses, Bonferroni corrections were applied to the standard *p* < 0.05 threshold. Samples showing a statistically significant effect were validated in an independent repeat under identical conditions. P values < 0.05 were considered statistically significant.

## Results

### Raisin extract

**Fig. 1:**
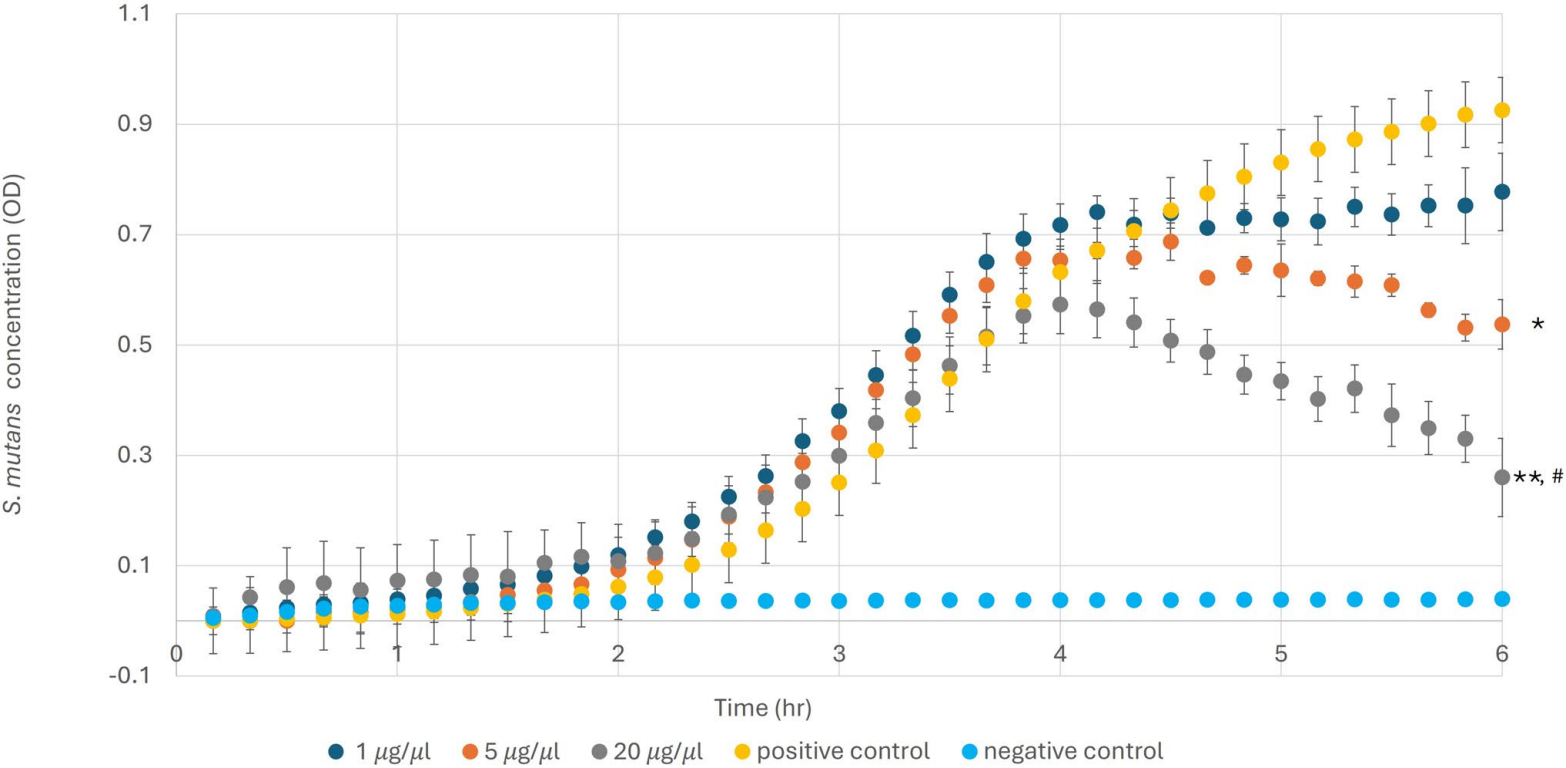
Inhibition of *S. mutans* growth by raisin extract 1–20 μg/μl. Water and chlorhexidine 0.2% served as the negative and positive controls, respectively. *p = 0.002 for the comparison between raisin extract 5 μg/μl and the negative control; ** p  < 0.001 for the comparison between raisin extract 20 μg/μl and the negative control; #p = 0.001 for the comparison between raisin extract 20 μg/μl and raisin extract 5 μg/μl. Each data point is the mean and standard deviation of three triplicates

5 μg/μl and 20 μg/μl statistically significantly inhibited the growth of *S. mutans* at 6 h compared to the negative control (*p* = 0.002 and *p* < 0.001, respectively). The inhibitory effect of the raisin extract 20 μg/μl was also statistically significantly higher than raisin extract 5 μg/μl (*p* = 0.001). No statistically significant inhibition of *S. mutans* growth by raisin extract 1 μg/μl compared to the positive control was observed at 6 h (Fig. 1).

### Pomegranate peel extract

**Fig. 2:**
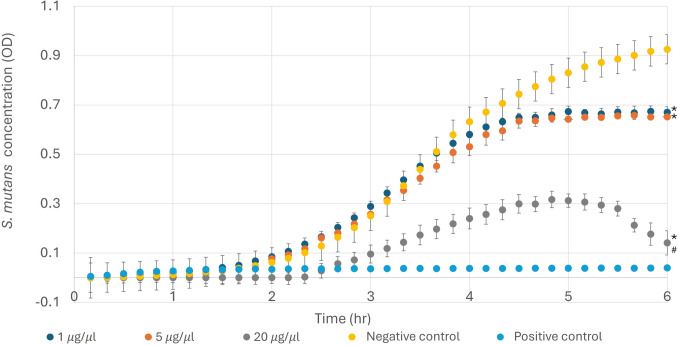
Inhibition of *S. mutans* growth by pomegranate peel extract 1–20 μg/μl. Water and chlorhexidine 0.2% served as the negative and positive controls, respectively. *p < 0.01 for the comparison between each pomegranate peel extract concentrations and the negative control; #p < 0.001 for the comparison between pomegranate peel extract 20 μg/μl and pomegranate peel extract 5 μg/μl. Each data point is the mean and standard deviation of three triplicates

1, 5, and 20 μg/μl statistically significantly inhibited the growth of *S. mutans* for up to 6 h compared to the negative control (*p* = 0.0015, *p* = 0.0307, and *p* = 0.0012, respectively). Additionally, the growth inhibition by pomegranate peel extract 20 μg/μl was statistically significantly higher than that of the 5 μg/μl concentration (*p* = 0.002), while no statistically significant difference in growth inhibition was observed between 1 μg/μl and 5 μg/μl (Fig. 2).

### Fresh pomegranate juice

**Fig. 3:**
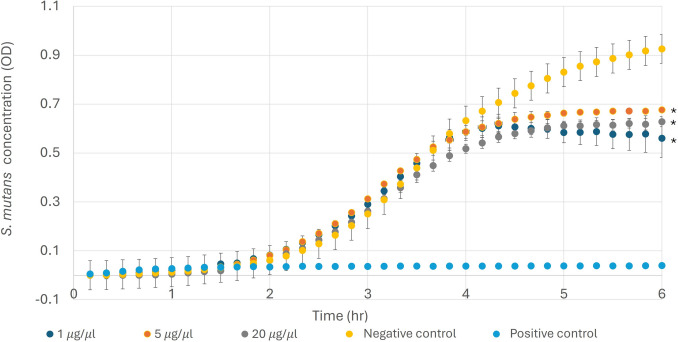
Inhibition of *S. mutans* growth by fresh pomegranate juice 1–20 μg/μl. Water and chlorhexidine 0.2% served as the negative and positive controls, respectively. *p < 0.05 for the comparison between pomegranate peel extract concentrations and the negative control. Each data point is the mean and standard deviation of three triplicates

1, 5, and 20 μg/μl statistically significantly inhibited *S. mutans* growth at 6 h compared to the negative control (*p* = 0.043, *p* = 0.005, *p* = 0.012, respectively). Growth inhibition was similar among the three juice concentrations, indicating that all concentrations demonstrated similar activity (Fig. 3).

### Cranberry extract

**Fig. 4:**
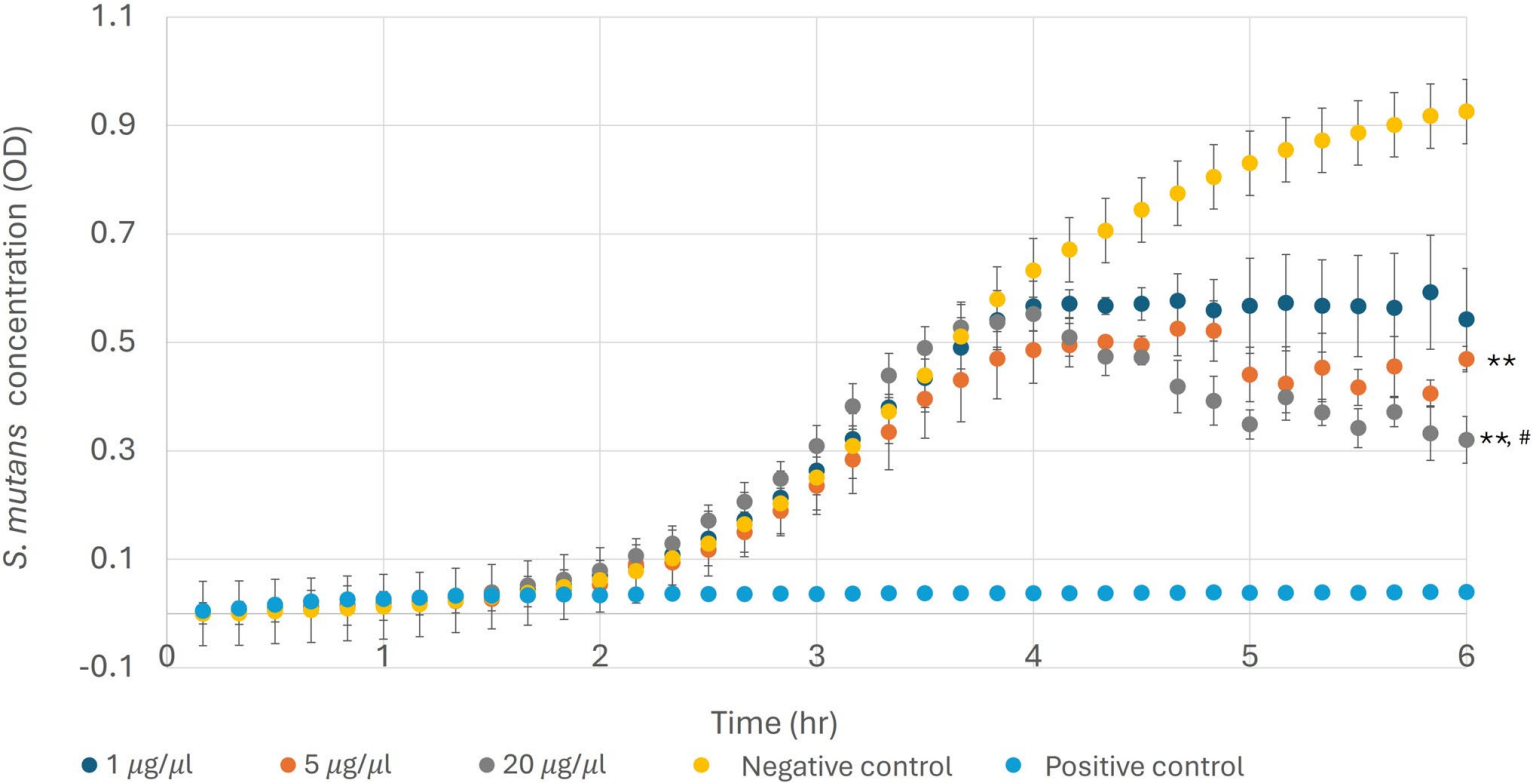
Inhibition of *S. mutans* growth by cranberry extract 1–20 μg/μl. Water and chlorhexidine 0.2% served as the negative and positive controls, respectively. **p < 0.001 for the comparison between cranberry extract concentrations and the negative control; #p = 0.003 for the comparison between cranberry extract 20 μg/μl and cranberry extract 5 μg/μl. Each data point is the mean and standard deviation of three triplicates

5 μg/μl and 20 μg/μl statistically significantly inhibited the growth of *S. mutans* at 6 h compared to the negative control (*p* = 0.0004 and *p* < 0.0001, respectively). The inhibitory effect of the cranberry extract 20 μg/μl was also statistically significantly higher compared to that of cranberry extract 5 μg/μl (p = 0.003). No statistically significant inhibition of *S. mutans* growth by cranberry extract 1 μg/μl compared to the negative control was observed at 6 h (Fig. 4).

### Cherry extract

**Fig. 5:**
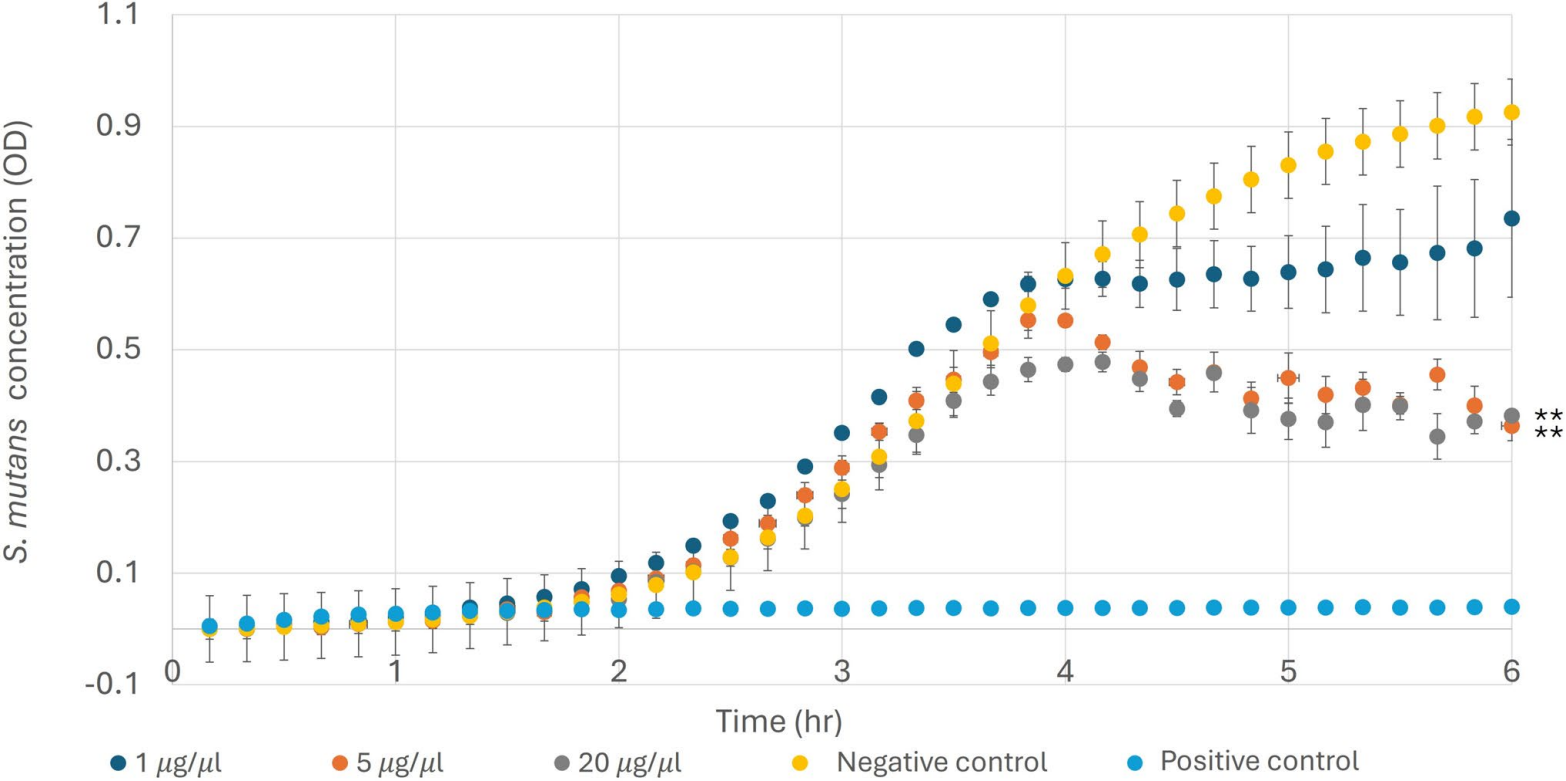
Inhibition of *S. mutans* growth by cherry extract 1–20 μg/μl. Water and chlorhexidine 0.2% served as the negative and positive controls, respectively. **p < 0.01 for the comparison between cherry extract concentrations and the negative control. Each data point is the mean and standard deviation of three triplicates

5 μg/μl and 20 μg/μl statistically significantly inhibited the growth of *S. mutans* at 6 h compared to the negative control (*p* = 0.008 and *p* = 0.007, respectively). No statistically significant inhibition of *S. mutans* growth by cherry extract 1 μg/μl compared to the negative control was observed at 6 h (Fig. 5).

**Fig. 6:**
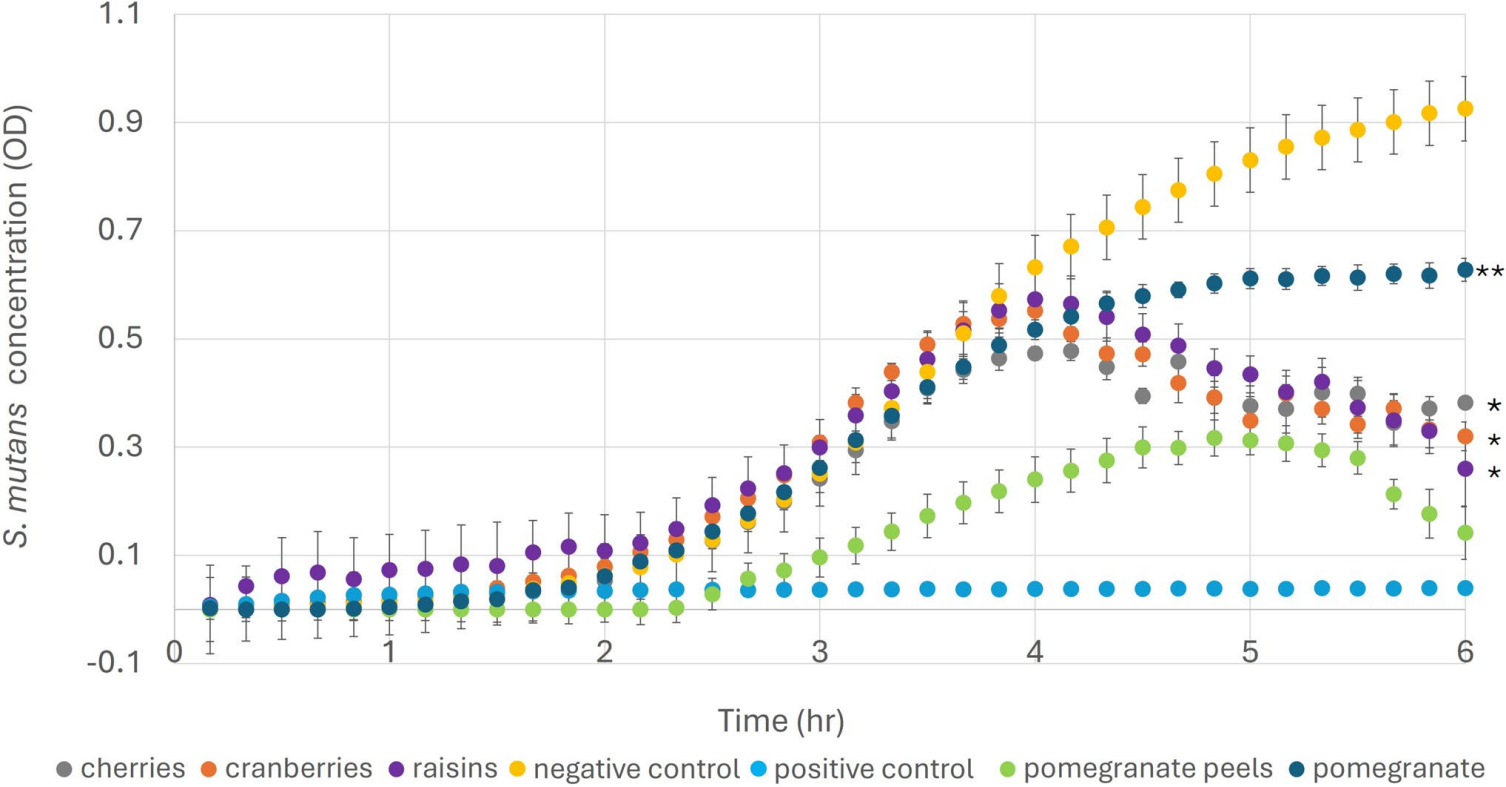
Comparison of the inhibition of *S. mutans* growth among fruit extracts 20 μg/μl *p < 0.05 for the comparison between pomegranate peel extract versus raisin extract, cranberry extract, and cherry extract; **p = 0.0005 for the comparison between pomegranate peel extract and pomegranate juice

Comparison among the highest concentration of all fruit juices showed that pomegranate peel extract 20 μg/μl showed the highest inhibition of *S*. *mutans* growth at 6 h compared to all fruit extracts tested at this concentration (*p*  = 0.046, *p* = 0.022, *p* = 0.017, and *p* = 0.0005 for the difference between pomegranate peel extract versus raisin extract, cranberry extract, cherry extract and pomegranate juice, respectively). No statistically significant difference in growth inhibition at 6 h was observed between peel extract 20 μg/μl and the positive control, chlorhexidine (Fig. 6), suggesting that this concentration has the same effectiveness in *S. mutans* growth inhibition as chlorhexidine.

## Discussion

The results of the present study have shown that extracts of dried raisins, cranberries, and cherries, as well as pomegranate peel extract and juice inhibited *S. mutans* growth in a dose-dependent manner. Statistically significant inhibition at 6 h compared to the negative control was observed for raisin, cranberry, and cherry extracts at 5 and 20 μg/μl, as well as for pomegranate peel extract and juice at 1 – 20 μg/μl. Among the highest concentrations tested, pomegranate peel extract (20 μg/μl) demonstrated the strongest inhibition, significantly surpassing raisin, cranberry, cherry, and pomegranate juice. Notably, its inhibitory effect did not differ from that of chlorhexidine, indicating that pomegranate peel extract at this concentration is as effective as the positive control. Our findings are in accordance with previous studies which found that red fruit can inhibit cariogenic bacteria growth, alone, and with other plant extracts (Amarowicz and Shahidi 2017; Ferrazzano et al. 2017; Sateriale et al. 2020).

Extracts of pomegranate peel contain high concentrations of hydrolyzable ellagitannins, glycosylated derivates of ellagic acid and free ellagic acid (Pagliarulo et al. 2016; Singh et al. 2023). Pomegranate juice is a source for phenolic compounds, particularly, anthocyanins hydrolyzable tannins (gallotannins), ellagitannins, gallagyl esters, hydroxybenzoic and hydroxycinnamic acid (Díaz-Mula et al. 2019). Ferrazzano et al. (2017) showed that pomegranate juice extracts effectively inhibit the growth and survival of *S. mutans* with a minimal inhibitory concentration of 10 μg/μl and a minimal bactericidal concentration of 15 μg/μl; also finding that pomegranate peel was more potent than juice. In line with Ferrazzano et al.’s findings, in the current study, pomegranate peel extract demonstrated a stronger and non-dose-dependent effect on bacterial growth compared to pomegranate juice. The effect of pomegranate peel extract at the highest concentration on inhibiting bacterial growth was comparable to the effect of chlorhexidine 0.2%, a known antibacterial agent that leads to the complete suppression of the bacterium (Jenkins et al. 1988; Karpiński and Szkaradkiewicz 2015), including bacteria in biofilm (Quintas et al. 2015). Pagliarulo et al. (2016) found that pomegranate peel extracts have much higher bacteriostatic/bactericidal activity than seeds, confirming our observations. In a double-blind cross-over clinical trial conducted among 14 children aged 8—10 years, whole pomegranate fruit extract and chlorhexidine 0.12% each statistically significantly decreased the oral hygiene index-simplified score by 34% and 36%, respectively (*P* < 0.05), with no significant difference between them (*P* > 0.05) (Mahd et al. 2023).

Cranberry is a particularly feasible and sustainable source of standardized bioactive compounds, as it is a chemically and genetically well-characterized fruit, with highly standardized methods for extracting its biologically active components (Philip and Walsh 2019). Cranberry mouthwash was found to be as effective as chlorhexidine 0.2% in decreasing *S. mutans* counts after 2 weeks of use by participants aged 18–20 years (Khairnar et al. 2015). In a single-center double-blind, parallel, non-inferiority clinical trial among 280 children aged 8–12 years, cranberry mouth rinse decreased *S. mutans* counts after one month of use and was non-inferior to fluoride mouth rinse used for the same period (Bansal et al. 2024). (Khairnar et al. 2015; Philip et al. 2020). In a parallel 3-group double-blinded randomized controlled trial among children and adolescents older than 10 years, dentifrices containing casein phosphopeptide-amorphous calcium phosphate supplemented with polyphenol-rich cranberry extracts beneficially modulated the microbial ecology of dental plaque, significantly decreasing *S. mutans* levels in a group of high caries-risk patients (Philip et al. 2020). A systematic review of 22 studies reported that cranberry extract 0.5 – 25 mg/mL was shown to inhibit the growth of *S. mutans*, reduce biofilm formation capacity, decrease polymicrobial biofilm biomass, buffer pH drop, and deregulate the expression of glycosyltransferases B and C (Castellanos et al. 2024).

The ability of raisins to inhibit *S. mutans* growth in a dose-dependent manner was also demonstrated in the present study. In the past, raisins were considered as fruit that encouraged the development of caries (Damle 2013); however, studies have demonstrated the excellent antibacterial and antioxidant capacities of raisins both in vitro and in vivo*,* which were attributed to its phenolic compounds, with the highest concentration found in grape seeds (Abouzeed et al. 2018; Jayaprakasha et al. 2003). In a randomized double-blind study, which compared the effect of mouthrinses containing chlorhexidine, grape seeds (*vitis vinifera*), pomegranate extract, and *Terminalia chebula* among 80 children 8—15 years of age, the mouthrinse containing grape seeds showed the greatest reduction in plaque index score after 16 days of use. The reduction in *S. mutans count* after 16 days was similar to the reduction in *S. mutans* observed following chlorhexidine mouthrinse use (Mishra et al. 2019). However, it should be noted that the observed reduction in *S. mutans* counts does not necessarily imply a reduction in caries experience as additional species or health conditions may also play a role in caries etiology (Dinis et al. 2022a).

Among the fruit extracts tested in the present study, cherries showed the least activity at the highest concentration. This finding contrasts with other studies. Homoki et al. (2018) showed that *S. mutans* levels decreased faster and to a greater extent in the presence of cherry extract. Ben Lagha et al. (2020) demonstrated that sour cherry extracts, even at low concentrations, significantly reduce the ability of *S. mutans* to form biofilms. The antibiofilm property of the cherry extracts has a large activity spectrum given their effects on fungi as well as Gram-positive and Gram-negative bacteria. It may be related to their ability to modify the microbial cell surface hydrophobicity, inhibit the gene expression of adhesins, or interfere with the quorum sensing. Because the adherence of oral microorganisms to tooth enamel surface is the primary step in dental plaque formation, this property may be regarded as promising in preventing caries (Ben Lagha et al. 2020).

## Limitations

It is important to acknowledge several limitations of this study. First, the concentrations of polyphenols in each fruit extract were not quantified. Future research should analyze the polyphenol content, identify the compounds most active against *S. mutans*, and assess whether any display synergistic effects. Furthermore, polyphenols’ antioxidant activity should be quantified and correlated with antimicrobial efficacy to further elucidate their mechanism of action against *S. mutans*. Second, although the fruit extracts were diluted in water to mimic the dilution by saliva during eating, this in vitro model does not account for additional factors in the oral environment such as host immune responses, salivary enzymes, or the complexity of biofilm formation, including interactions with other microorganisms. Third, the effect of chlorhexidine 0.2% on *S. mutans* was used as a positive control to the fruit extracts. Although chlorhexidine mouthwashes have demonstrated significant reductions in *S. mutans counts*, direct clinical benefits, such as caries reduction, have not been proven for chlorhexidine (Poppolo Deus and Ouanounou 2022; Walsh et al. 2015). Last, spectrophotometric measurements reflect only planktonic growth within the wells and do not capture bacterial cells adhering to the polystyrene surface. Since a substantial portion of growth occurs within the biofilm and above the adhesion layer, and given that we did not examine the initial colonization stage, which also depends on the acquired pellicle, using polystyrene wells was considered an appropriate approach for this model.

## Conclusions

This study evaluated the acute effects of specific fruit extracts on *S. mutans* growth. We have shown that extracts of raisins, pomegranate juice and peels, cranberries, and cherries inhibit the growth of *S. mutans* in a dose-dependent manner for up to 6 h in vitro, with pomegranate peel extract showing the greatest potency. Given that the water-soluble compounds in these fruit extracts have transient oral exposure—whether from food consumption or oral hygiene products—the 6-h experimental window adequately captures their short-term bacteriostatic activity. Due to the in vitro design of the study, further studies are needed to evaluate these effects in the oral environment, to determine the most effective doses and to evaluate whether the cariostatic effects can actually translate into preventing dental caries in high-risk individuals and population groups.

## Data Availability

The data supporting the findings in this study are available from the corresponding author upon reasonable request.
